# High Density Crossbar Arrays with Sub- 15 nm Single Cells via Liftoff Process Only

**DOI:** 10.1038/srep32614

**Published:** 2016-09-02

**Authors:** Ali Khiat, Peter Ayliffe, Themistoklis Prodromakis

**Affiliations:** 1 Nanoelectronics and Nanotechnology Research Group, Department of Electronics and Computer Science, Faculty of Physical Science and Engineering, University of Southampton, University Road, SO17 1BJ, Southampton, United Kingdom; 2 Southampton Nanofabrication Centre, University of Southampton, Highfield Campus, Southampton SO17 1BJ, UK

## Abstract

Emerging nano-scale technologies are pushing the fabrication boundaries at their limits, for leveraging an even higher density of nano-devices towards reaching 4F^2^/cell footprint in 3D arrays. Here, we study the liftoff process limits to achieve extreme dense nanowires while ensuring preservation of thin film quality. The proposed method is optimized for attaining a multiple layer fabrication to reliably achieve 3D nano-device stacks of 32 × 32 nanowire arrays across 6-inch wafer, using electron beam lithography at 100 kV and polymethyl methacrylate (PMMA) resist at different thicknesses. The resist thickness and its geometric profile after development were identified to be the major limiting factors, and suggestions for addressing these issues are provided. Multiple layers were successfully achieved to fabricate arrays of 1 Ki cells that have sub- 15 nm nanowires distant by 28 nm across 6-inch wafer.

Emerging non-CMOS technologies such as crossed semiconductor nanowire field-effect transistor (cNW-FET) arrays^1^ and memristors^2^,^3^ although showing promising results at single device level, have not yet been able to exploit their full potential in density integration. The most effective architecture to generate the highest possible density consists of crossbar arrays of metal nanowires, where multiple patterns of individual layers are needed. This extreme scalability results on a footprint of 4 F^2^/cell in 2D that would be stackable to achieve 3D arrays. This work particularly exploits the scaling limits of high density nanowire arrays for memristor applications; these are based on two terminal devices that rely on metal-insulator-metal (MIM) architecture. Their promising characteristics can be leveraged for resistive random access memory devices (RRAM), neuromorphic computing and sensors. All the aforementioned applications are particularly interested in exploiting the technology’s scalability while complying with CMOS integration strategies.

Previous studies have shown how to improve the resolution of the patterned features by using electron beam (e-beam) lithography technology. Chen and Ahmed^4^ demonstrated that achieving 10 nm features could be possible using an ultrasonic agitation during resist development, avoiding intermolecular forces that were preventing the dissolution of the exposed resist. The current state-of-the-art was reported by^5^, where lower molecular weight resist and a poor solvent developer system were used to achieve minimum feature size of 4 nm. Mohammad *et al.*^6^ proposed a process for obtaining small and dense features by combining low-voltage exposure and cold development. 15 nm features at 40 nm pitch were reached after resist development, while Cord *et al.*^7^ have optimized the development temperature of PMMA which allowed obtaining sub- 10 nm features with 60 nm pitch in 1 × 1 μm^2^ area.

Even though several studies have succeeded in obtaining small and dense features, only a few of them have utilized efficiently liftoff to obtain continuous metal nanowires. In this study, we deliberately avoid the use of etching for minimizing potential side effects that lead into the mechanical or chemical compromising of the deposited thin films and their interfaces. Dial *et al.*^8^ have reported fabrication of 20 nm Gold lines with 60 nm pitch, while Craighead *et al.*^9^ have succeeded fabricating 20 nm wide, 15 nm thick and 70 nm pitch metal lines. Although smaller features were sporadically obtained there are no relevant reports on continuous metal nanowires below this threshold. Similar results were also demonstrated by others when they attempted to reduce the nanowire widths and the obtained metal lines after liftoff become granular. For example, 4 to 8 nm granular metal islands were demonstrated in references^10^,^11^,^12^,^13^. Vieu *et al.*^10^ have also reported achieving 20 nm width, 10 nm thick and 40 nm pitch metal nanowires and Mohammad *et al.*^14^ have succeeded in producing sub- 20 nm wide, 12 nm thick and 50 nm pitch Chromium lines after cold development at −15 °C and liftoff process. The aforementioned studies are all limited to single layer patterning.

Various parameters have been identified to have an impact on the successful development of nanowires purely via liftoff process, while Lee *et al.*^15^ reporting an array of 30 × 30 nm^2^ cross-points with 150 nm spacing. This study employed a double layer resist (ZEP 520A7/LOR 1A) for respectively defining the features and aiding the liftoff process. Moreover, it was demonstrated in reference^16^ that 40 × 40 metal arrays of about 50 nm width can be fabricated at 100 nm pitch. Clearly, the larger the array, and consequently area of development, the more difficult liftoff uniformity becomes.

Nano-imprint lithography (NIL) is an alternative technology, which was recently employed for fabricating high density arrays for RRAM application^17^,^18^,^19^. So far the smallest reported devices comprise 8 nm wide electrodes, however, only 100 nm pitch arrays were demonstrated^20^. Yet, this technology imposes additional challenges as it requires e-beam lithography for patterning the mask masters and additional reactive ion (RIE) and wet etching steps for further reducing the attained size. Moreover, the achieved reported yield of devices was relatively low, despite the fact that the masters were constrained into relatively small area, i.e. 1 × 1 inch^2^. Arrays with 18 nm pitch were also reported in^21^, where authors adopted nano-injection lithography which uses e-beam-assisted chemical reaction to deposit the electrodes^22^. The attained throughput for large area patterning is however low.

Here, we demonstrate fabrication method for achieving nanowire arrays of high-density across 6-inch wafer based on liftoff process following e-beam lithography, evaporation and sputtering. Dense, thick and concurrently of minimum feature nanowires were achieved through a double layer liftoff process. Ultrasonic agitation was avoided for minimizing potential irreversible damage of the nanowires and the layers underneath fabricated in the preceding steps. Our method further enables the fabrication of planar nano-devices due to the attained control on reducing the gaps between nannowires. In this work we tackle the issue of nanowire fabrication in three stages: In stage 1 we demonstrate fabrication of single cross-points nanowires (SCnW) and array cross-point nanowires (ACnW). In stage 2 we concentrate on nanowire width scaling and present the fabrication methods used to reduce nanowire sizes down to 15 nm. Finally, in stage 3 we increase the overall density of ACnW by focusing on reducing the pitch between nanowires.

## Results

### Fabrication of single and array cross-points nanowires

**Figure 1:**
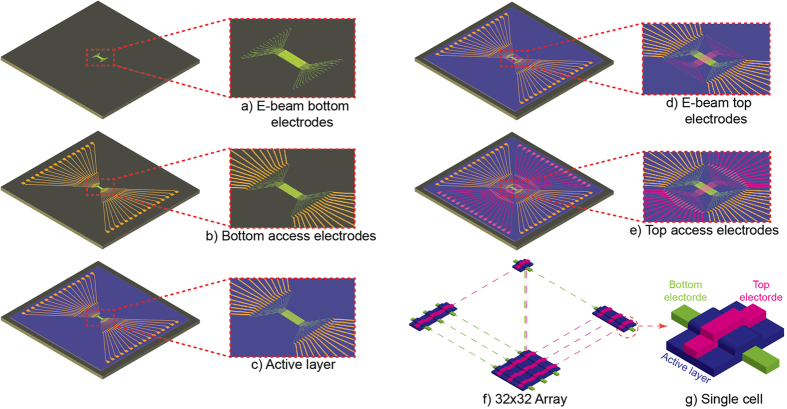
Main fabrication steps of dense nano-scale array of RRAM devices. (**a**) E-beam bottom electrodes, (**b**) bottom access-electrodes, (**c**) TiO_2−x_ active layer, (**d**) e-beam top electrodes, (**e**) top access-electrodes, (**f**) 32 × 32 ACnWs, (**g**) schematic representation of a single RRAM cell.

Although our approach can be exploited broadly in emerging nano-electronic concepts, throughout this work we delineate our methodology in relation to RRAM nano-devices that belong to the family of memristors. Such elements are based on MIM architecture with cross-points nanowires configuration enabling the densest topology. Our prototypes, illustrating our approach, were fabricated on 6-inch wafers using a set of masks to pattern and duplicate the devices across the wafer. Two main architectures were adopted in this process; single cross-points nanowires and array of cross-points nanowires. The main fabrication steps are represented in Fig. 1, with Fig. 1f representing the schematic of 32 × 32 ACnWs and Fig. 1g a single cell. Considering the die size of 3 × 3 mm^2^, which contains 9 × (32 × 32) arrays, 184 dies were fabricated each time across 6-inch wafer; 92 in CB and 92 in SA configurations.

In order to demonstrate nanowire fabrication (stage 1) we employed a process using double layer resist MMA/PMMA495 which was exposed by e-beam at 100 kV, 1 nA and 60 micron aperture, at a dose of 450 μC/cm^2^.

**Figure 2:**
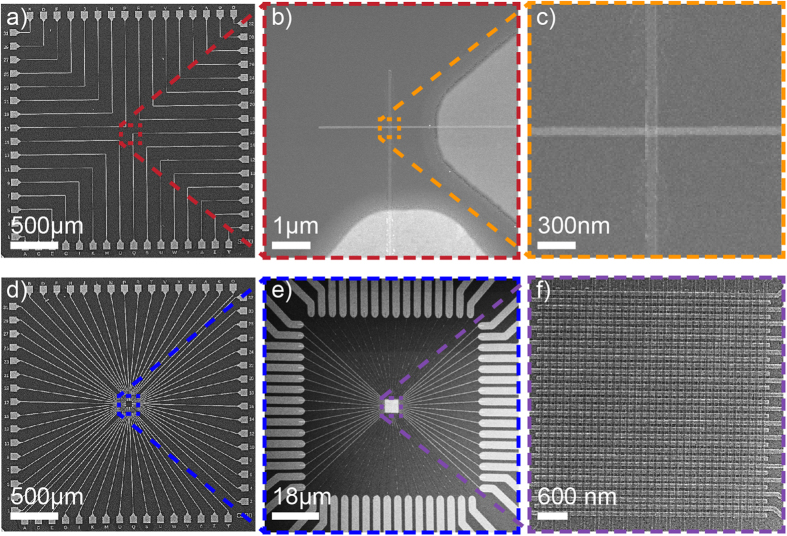
SEM images of (**a**) SCnWs with 70 nm widths, and (**b**,**c**) show zoom-in images of the corresponding areas. (**d**) ACnWs with widths and gaps of 70 nm, (**e**,**f**) show zoom-in images of the corresponding areas.

The fabricated devices were imaged by a JEOL 7500F scanning electron microscope (SEM). Figure 2 shows examples of the fabricated devices for the two configurations, SCnW (32 single cells) and ACnW (an array of 32 × 32 nanowires = 1 Ki cells), with 70 nm wide nanowires. One can distinguish the top and bottom access-electrodes, and the nanowires’ top and bottom electrodes (TE, BE) which are magnified in the insets, for better clarity. Different nanowire dimensions were obtained from 200 nm down to 70 nm with a minimum achieved gap of 70 nm.

### Reducing the nanowire size to sub- 15 nm features

In the nanowire width reduction stage (stage 2) single layer resist PMMA950 A4 was used to achieve even finer features by liftoff process thanks to its higher molecular weight, which slows the development and improves its contrast. This resist has the characteristics of generating clear negative side-wall slopes that allow good liftoff quality. It was exposed to e-beam with a dose of 1000 μC/cm^2^ using proximity corrections, 100 kV, 1 nA and 60 micron aperture. This generates a spot size of approximately 4.7 nm in diameter. Doses on the range 800–1000 μC/cm^2^ were found to be optimum for our purposes. The resist was then developed in a cold developer^11^,^12^ using MIBK:IPA (1:1) at 5 °C for 60 seconds and the reaction was stopped by immersing the wafer in IPA for 30 seconds. Back scattering, secondary electrons and use of the same dose and proximity corrections for both BEs and TEs required a slightly longer development time, of 5 seconds, for TEs of comparable dimensions. Under these conditions, the resist doesn’t crosslink, thus enabling opening the patterned features for liftoff process. This process was used without sonication at all stages to avoid damage on the previously deposited films and to sustain the miniscule metal wires in position, thus ensuring continuity along the desired nanowire length. One line of 4.7 nm shots in diameter suffices for opening sub- 10 nm features in the resist. Nonetheless, various resist residues could remain on the developed areas, requiring slightly longer development time for ensuring a perfectly clean surface that is essential for good liftoff at this scale. This however occurs at the expense of widening the developed features. Chen and Ahmed^4^ have used ultrasonic agitation during development to help dissolving the exposed resist by breaking the intermolecular forces. Nonetheless, their approach didn’t yield reliably continuous metallic nanowires.

**Figure 3:**
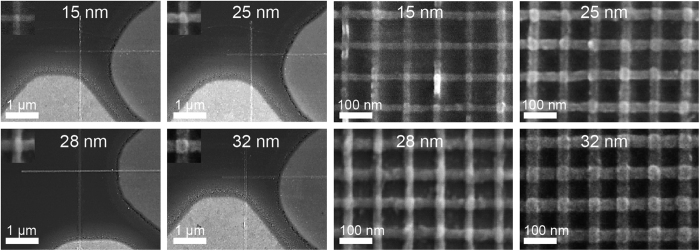
Reducing the features’ sizes. Single cross-point nanowires (SCnW): (**a**) 15 nm, (**b**) 25 nm, (**c**) 28 nm and (**d**) 32 nm with the insets depicting the crossbars, scale bars are 1 μm. Array of cross-point nanowires (ACnW) at 100 nm pitches: (**e**) 15 nm, (**f**) 25 nm, (**g**) 28 nm and (**h**) 32 nm, scale bars are 100 nm.

Instead of having single 4.7 nm shots we further optimized our process for two shots overlapping slightly (by 15%), along the exposed areas within the width of the lines. This allowed having a higher dose in the center and homogenous exposure at the extremes. Development was found to commence from the center and with time extending to the sides without leaving any residues at the surface. As the back scattering and secondary electrons affect the exposed areas, the smallest achieved lines via this approach were in the range of 10–15 nm and following metal evaporation and liftoff, the smallest metallic nanowires obtained successfully were in the range of 10–15 nm; with the 15 nm features more reliably obtained. Nanowires with various widths: 15 nm, 25 nm, 28 nm and 32 nm are shown in Fig. 3 for SCnW and ACnW with 100 nm pitches.

For the ACnW configuration, the smallest gap between successive nanowires that rendered a 100% yield of this process across 6-inch wafer was found to be in the range 70–85 nm; for line widths varying from 200 nm down to 20 nm. The yield drops to 55% for 15 nm wide wires. The yield is calculated based on the number of good nanowires checked by SEM (in term of continuity) in 6 dies across 6 inch wafer, with each die containing 32 BE and 32 TE nanowires. This gives a yield-figure calculated based on 1152 nanowires.

Clearly, the gap between successive nanowires is a critical parameter and reducing it makes effective nanowires development more challenging. After a slight over development, the patterned features in the resist tend to collapse due to the capillary force and the undercut on the resist side-walls that were enforced by the back scattering and secondary electrons, eventually resulting into the features merging. Optimizing the development process allowed us to further reduce the separating gap between nanowires; for example to about 50 nm for 50 × 50 nm^2^ cross-point arrays by a careful and gentle development and drying. Nonetheless, this had affected obtaining reliably the smallest features (15 nm) developed within the same wafer, due to resist residues remaining on the surface that caused liftoff to fail. Introducing auxiliary process steps such as RIE or increasing development time would have negative impact either on the nanowire gap separation (for example merging the features) or on the smallest attainable features. It’s worth mentioning that this effect occurred within the same exposed layer and any additional film to the device would alter the overall development conditions. Yet, this effect is limited within the new layer. The residual resist remained on the surface after liftoff can be removed by a much longer liftoff time.

### Increasing the density of cross-points nanowires array

One can further reduce the resist thickness to avoid collapsing the patterned resist by reducing the undercuts and the surface tension during development, resulting in reducing the gaps between adjacent nanowires (stage 3). The thinner the resist the lower the influence of back scattering and secondary electrons becomes. Diluting the resist in anisole reduces its viscosity and in consequence its thickness, for the same spinning conditions. We have considered four different thicknesses to optimize the gap reduction by liftoff process: 1:1, 1:1.5, 1:2 and 1:3 volume ratios of PMMA:Anisole (ZEP A) that resulted in 50 nm, 40 nm, 30 nm and 15 nm thick resist layers, respectively. After resist development with a cold developer, 1:1 (MIBK:IPA) at −15 °C^6^,^7^, the ratio 1:3 was found to give the narrawest gap. Gap of 15 nm resulted from 1:1.5, 1:2 and 1:3 diluted resists.

**Figure 4:**
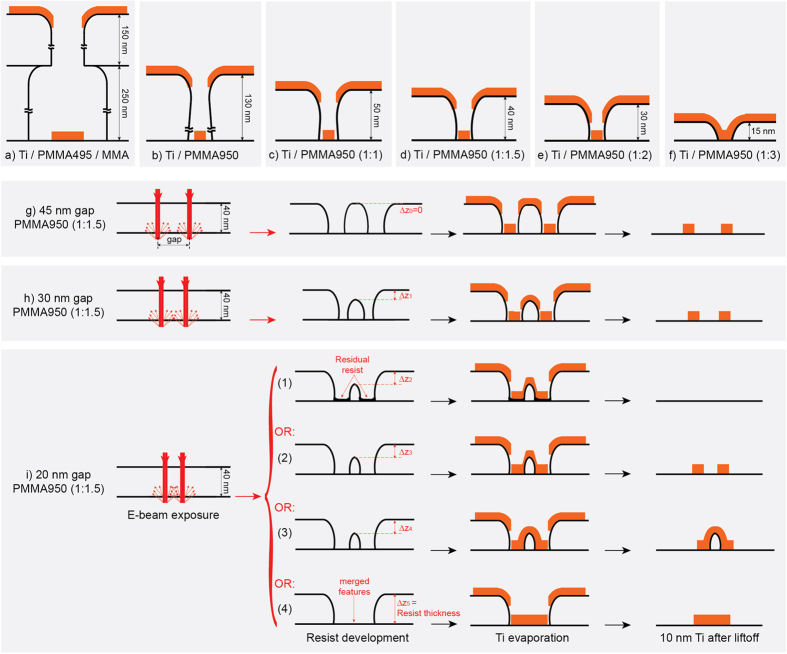
Resist limitations. Influence of the resist on the liftoff quality of 10 nm Ti. (**a**) PMMA495/MMA (150/250 nm), (**b**) PMMA950 (130 nm), (**c**) PMMA950:ZEP A (50 nm), (**d**) PMMA950:ZEP A (40 nm), (**e**) PMMA950:ZEP A (30 nm) and (**f**) PMMA950:ZEP A (15 nm). Influence of the exposure gaps in PMMA950:ZEP A (1:1.5) resist on the nanowire density; (**g**) 45 nm, (**h**) 30 nm and (**i**) 20 nm gaps.

It’s interesting to note that diluting the resist affects the undercut after resist development due to its thickness reduction. This also further reduces the feature size after development; thus, liftoff process becomes challenging. Figure 4a–e shows schematic cross-sections of all the used configurations after metal deposition. The MMA/PMMA495 method was found to give good results, however, small and dense features were obtained using PMMA950 A4 resist. After development, the radius of the features’ edges is about 20 nm, therefore, a 15 nm thick resist does not allow having the undercuts that enable a good liftoff. On the contrary, resists with thicknesses of 40 nm, 30 nm, 50 nm and 130 nm (higher than 20 nm) support undercuts after development which resulted into good liftoff, thus continuous nanowires were achieved.

We have observed that after liftoff the sub- 15 nm nanowires break easily at the angles (Figure S6), resulting in low yields (about 55%). Therefore, we have reduced the shot step to 1 nm (75% shots overlaps) and the beam current to 100 pA (spot size becomes 4 nm), increasing the yield to 95%.

## Discussion

It was still possible to obtain nanowires with the 15 nm thick resist when sonication was used, nonetheless, discontinuities and side-walls’ fences were generated. It’s important mentioning that during metal evaporation the temperature of single grains varies with the chosen metal because of the difference in their melting points. This would slightly affects the 20 nm radius of the features’ edges (resist edges), which in terns affects liftoff quality. In extreme cases, this would also precipitate resist collapsing.

A good liftoff in ACnWs is strongly related to the nanowires pitch but also to the lengths of the nanowires that becomes even more challenging for thin resists. Nanowires are typically obtained when they are far apart as the solvent can more easily penetrates underneath the metal creating a force for achieving liftoff. This liftoff force is proportional to the gap width and inversely proportional to the nanowires’ length. A direct consequence of this relation is the possible presence of fences on the nanowires, which is not ideal for the devices. Although, our approach was able to demonstrate reliable fabrication of 15 nm cells in ACnWs, sub- 10 nm nanowires were also obtained but at lower yields. It appears that the size of the features after development is limited to this range. We argue that this is due to inhomogeneous breaking of polymer chains in the resist. 15 nm gaps were achieved after liftoff. However, in this occasion, the nanowire lengths were less than 0.5 μm. Reproducing the device’s configuration shown in Fig. 2 but in higher density requires fabricating 32 × 32 nanowires of 15 nm widths with the smallest possible gaps. This would cover a surface area larger than 1 × 1 μm^2^. Consequently, further optimizations were necessary to fabricate the smallest and densest nanowires, with 1 Ki cells.

Towards identifying our process limits, 4 resist configurations were adopted: not-diluted, 1:1, 1:1.5, 1:2 and 1:3 diluted resists. Schematics of their profiles are presented in Fig. 4b–f, respectively. For a complete picture, we have also added the resist profile of the double layer resist PMMA/MMA (Fig. 4a). All the configurations result in good liftoff except the case presented in Fig. 4(f) where the resist is too thin and creating negative slope in the resist profile after development becomes not possible. 8 samples were prepared for each configuration and were developed at different times in either 1:3 (MIBK:IPA) or 1:2 (MIBK:IPA). The highest density ACnWs achieved reliably were obtained with 1:1.5 diluted resist, developed with 1:3 (MIBK:IPA) cold developer at −15 °C for 120 seconds then in IPA for 30 seconds. The smallest achieved nanowires are of 14 ± 2 nm widths and the smallest gaps were of 28 ± 2 nm. Figure 4g–i shows schematics representing the gap influence on the nanowire density using 1:1.5 diluted resist, which is 40 nm thick; On one hand the back scattering and secondary electrons affect less the larger gaps than the smaller ones, as shown in Fig. 4g,h that represent good liftoff with gaps of 45 nm and 30 nm, respectively (see Figure S4a,b for the corresponding fabricated nanowires). On the other hand, when the gap is less than 40 nm the resist thickness in the dense area reduces during development (represented by ΔZ1 in Fig. 4h) because of the influence of the ~20 nm radius of the features’ edges. The bigger ΔZ the smaller is the undercut, consequently, the worse liftoff becomes. Figure 4i shows the method’s limit where the result is not consistent. After resist development, some areas are underdeveloped (Fig. 4(i1)), where residual resist remains on the surface leading to a complete liftoff even the nanowires, other areas are well-developed (Fig. 4i(2)) and others are merged (Fig. 4i(4)) thus bad liftoff occurs in this case as well. In the developed areas some parts show successful liftoff (Fig. 4i(2)) but others not (Fig. 4i(3)). We argue that this is happening because of thinning the resist (ΔZ3, ΔZ4 and ΔZ5 indicated in Fig. 4) and losing its negative slope’s aspect, after development. Consequently, repeatably good results are too critical and difficult to obtain (see Figure S5 in supplementary materials for more details). Therefore, the efficient gap to achieve reliable and reproducible arrays was found to be of 28 ± 2 nm (Fig. 4h), using 40 nm thick diluted resist.

Having metal nanowires with the lowest line resistances is very important for accessing emerging electron devices. To that end, various metal thicknesses were deposited on the smallest features then lifted-off to obtain the thickest possible nanowires. A 1:1.5 diluted resist resulted on a thickness of about 10 nm and if a short sonication is used 15 nm to 20 nm can be achieved. Not diluting the resist results into thicker film after spinning (130 nm). This enables obtaining thicker metal nanowires by liftoff; around 45 nm that can even be increased to 60 nm after a short sonication, at the risk of breaking the nanowires. Clearly, using double layer resists MMA/PMMA (250 nm/150 nm) permits having much thicker metal nanowires, about 100 nm, but to the detriment of their widths/gaps (minimum of 70 nm/70 nm).

**Figure 5:**
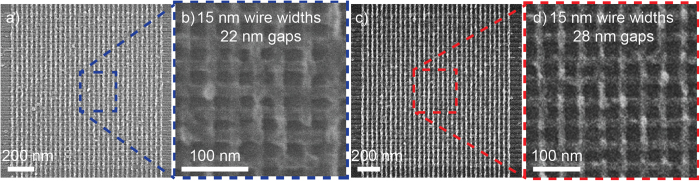
The optimum array cross-points devices with the smallest, densest and thickest metal nanowires. (**a**) 32 × 32 wires with 15 nm widths and 22 nm gaps (close-up image in **b**), (**c**) 32 × 32 wires with 15 nm widths and 28 nm gaps (close-up image in **d**).

Once the thickness, size and gap of the nanowires were optimized, RRAM cross-points array devices were fabricated (Fig. 5) following the steps represented in Fig. 1, without using any ultrasonic agitation. Figure 5a (close-up image is shown in Fig. 5b) shows an example of successful 32 × 32 ACnW with 15 nm wires and 22 nm gaps, however, with a limited reproducibility. In the other hand, Fig. 5c shows 32 × 32 ACnW (1 Ki cells) RRAM devices of 15 × 15 nm^2^ cross-points distant by gaps of 28 nm (close-up image is shown in Fig. 5d), highly reproducible. Taking into account the maximum clock of the e-beam tool, which is 50 MHz, writing time (exposure time) to pattern 32(TE) x 32(BE) nanowires is 2× (3 min and 47 seconds).

Devices, from 200 nm down to 15 nm, across 6-inch wafer were fabricated with a stack constituted of Ti/TiO_2−x_/Ti (10 nm/10 nm/10 nm). Although the yield is extremely high, achieving 95% across the full 6-inch wafer needs further optimization. It’s worth mentioning that the smaller the devices the more influential the variance becomes in term of width, line straightness and their 3D profiles; causing divergence from the original designs. Electrical characterisation results of these RRAM devices are shown in supplementary materials (Figures S1, S2 and S3).

Finally, the presented method allowed fabricating planar devices with sub- 18 nm gaps (Figure S6b,c). These devices were obtained with single e-beam lithography, metal evaporation and liftoff processes.

In summary, this work explored the scaling limits of the e-beam lithography using conventional resists, in liftoff configuration only. Even though transferring e-beam written features can be realized via various techniques, liftoff remains the less-invasive process that suits most emerging technologies’ needs. In addition, although smaller feature sizes have been demonstrated for single layer devices, high-density cross-bar arrays are yet to be developed. The proposed method was successful in achieving very high yield in 6-inch wafer using either double layer resists PMMA495/MMA or single resist PMMA950 A4. We have demonstrated the limitations of thinning PMMA950 A4 resist for achieving successful liftoff; the thickness has to exceed 20 nm and the metallization has to be considered carefully, in terms of temperature, thickness and material. Thin resists makes depositing some materials challenging due to their high melting temperatures and their large grain sizes. Thicker resists makes depositing thick metals possible; however, thin enough resists results in higher densities. Moreover, we have demonstrated successful liftoff in multiple layers that allowed fabricating 32 × 32 cross-points arrays of sub- 15 nm nanowires with 28 nm gaps across 6-inch wafer at very high yields, approaching 95%. We have also demonstrated similar arrays with 22 nm gaps, however, at lower yield. Finally, planar devices with 18 nm gaps using liftoff process only, of 10 nm thick Ti film, were demonstrated.

## Methods Summary

### Fabrication

#### Flowchart

All the devices exploited in this work were fabricated according the following flowchart; The 6″ wafer was first thermally oxidized to grow 200 nm SiO_2_, which serves as an insulating layer. Then, direct write e-beam lithography (JEOL JBX 9300FS) was employed to pattern the bottom electrode (BE) nano-structures. 10 nm Tatanium (Ti) was then evaporated and lifted-off (Fig. 1a). In turn, optical lithography was used to pattern the bottom access-electrodes, which were achieved after evaporating a 5 nm thick Ti and 25 nm Gold (Au) followed by liftoff process (Fig. 1b). Access-electrodes connect the pads to the nanowires, limiting the use of e-beam lithography in small areas, to overall reduce the writing time. Using optical lithography, reactive sputtering and liftoff process, a TiO_2−x_ (x = 0.05) active layer was patterned and defined, with a 10.0 ± 0.1 nm thickness achieved across the wafer through a Leybold Helios Pro XL Sputterer (Fig. 1c). The film was sputtered using a Ti metal target with 10 sccm flow of O_2_, 40 sccm Ar, 2 kW at the cathode, and 15 sccm O_2_, 2 kW at an additional plasma source for achieving near stoichiometric film quality. Next, 10 nm Ti top electrode (TE) nanometre features (Fig. 1d) and 25 nm top access-electrodes (Fig. 1e) were obtained in a similar fashion to the BEs and to the bottom access-electrodes, respectively. O_2_ plasma, at low power (100 W), was used after each lithography and liftoff steps to remove any non-desirable remaining resist on the surface.

### Liftoff processes

#### Nanowires

In this study, liftoff was used without any ultrasonic step for many reasons: as nanowire widths decrease, the combination of inherent nanowire material strength and adhesion force that help maintain structural integrity weaken. As a result, excessively thin nanowires become fragile and may be broken easily under ultrasonic agitation. Furthermore, at these nanometre dimensions inherent nanowire material strength is also compromised by the fact that the wires are constituted of few metal grains. It is still possible to obtain some successful devices, however, not reliably and, particularly, not across large surfaces such as a 6-inch wafer. In addition, sonication is generally used to help liftoff process because of the absence of negative slopes in the resist after development. This doesn’t allow having discontinuity of the metal between the patterned and non-patterned areas, which causes lifting of the nanowires in the bad adhesion areas and having fences in the good ones; fences that generates weak spots in the subsequent layers and ultimately in the devices. Particular attention and effort was made during fabrication process to create negative slopes and, therefore, avoid fences and at the same time creates discontinuity during metal deposition between the patterned and non-patterned areas in the resist. Consequently, evaporation process, using e-beam evaporation tool (Leybold_Lab700eb) that has high (1.5 m) crucible-to-wafer distance, gives an optimum result. Moreover, the deposited metal has a slightly positive side-wall profile generated by the metal deposition in the resist edges which then generates a shadowing effect (see the illustration in Figure S7). The resulted metal nanowire profile facilitates then the subsequent layer deposition, which would have a continuous profile even when thinner layers, compared to metal nanowires, are deposited. Thus, a complete insolation of the bottom electrodes is obtained in our devices. Liftoff process is done by putting the wafer in 1-Methyl-2-pyrrolidon (NMP) at room temperature and agitating it gently at the beginning to remove lifted metal from the small patterned areas then leave it soak for at least 12 hours to allow resist dissolving in the large non-patterned areas, and at the same time remove any residual resist from the wafer surface, particularly next to the nanowires. To finish liftoff process further agitation was carried out to remove the remaining metal from the wafer surface, and then it was rinsed and dried with water and N_2_, successively. Finally, to guarantee removing all resist residues, 60 seconds weak O_2_ plasma at 100 watts was applied.

#### Access-electrodes

Large features were lifted-off in NMP overnight, cleaned with water then dried with N_2_.

#### TiO_2−x_

TiO_2−x_ liftoff was done to create opening in the Pads and allow having access to the electrodes during electrical measurements. Optical lithography and negative resist was used to create negative slopes and facilitate liftoff, however, sputtering process deposit TiO_2−x_ everywhere even in the resist sidewalls, which makes liftoff process challenging, therefore, liftoff equipment “Optiwet-ST30” was used. This employs a beam of NMP, heated at 60 °C, followed by water jet for cleaning and N_2_ for drying at a pressure of 3 mbar. This method allows achieving good liftoff in the pads areas. It is worth mentioning that the nanowires are covered by the TiO_2−x_ film that protects them against water jet and N_2_ flow, otherwise many of the nanowires would be destroyed, affecting the overall attaining yield. This step is flowed by low power, 100 watts, RIE O_2_ plasma for 60 seconds to remove any residual resist remaining on the wafer surface.

### Electrical characterizations

The electrical characterization of our prototypes was carried out via DC and pulsing modes. The TE was biased and the BE was kept grounded for all the measurements.

#### DC mode

Keithley SCS-4200 instrument was used with Wentworth Laboratories AVT 702 semi-automatic prober to characterize the devices in DC sweeping mode (Figures S1 and S2).

#### Pulsing mode

Custom made electronic hardware with an mBED LPC1768 microcontroller board^23^ was used to characterize the devices in pulsing mode (Figure S3). This instrument is capable of addressing RRAM arrays up to 1 kb in size (32 × 32 cells).

### Data availability

The data for this paper can be found at: http://dx.doi.org/10.5258/SOTON/397940.

## Additional Information

**How to cite this article**: Khiat, A. *et al.* High Density Crossbar Arrays with Sub- 15 nm Single Cells via Liftoff Process Only. *Sci. Rep.* **6**, 32614; doi: 10.1038/srep32614 (2016).

## Supplementary Material

Supplementary Information
