# Supplementary material for: High Density Crossbar Arrays with Sub- 15 nm Single Cells via Liftoff Process Only
Source: Sci Rep. 2016 Sep 2;6:32614. doi: 10.1038/srep32614 (PMC5009344; doi:10.1038/srep32614)
Supplement: Supplementary Information [file srep32614-s1.doc]

**Supplementary information:**

High Density Crossbar Arrays with Sub- 15 nm Single Cells via Liftoff Process Only

Ali Khiat^*,a,b^, Peter Ayliffe^b^ and Themistoklis Prodromakis^a,b^

^a^Nanoelectronics and Nanotechnology Research Group, Department of Electronics and Computer Science, Faculty of Physical Science and Engineering, University of Southampton, University Road, SO17 1BJ, Southampton, United Kingdom.

^b^Southampton Nanofabrication Centre, University of Southampton, Highfield Campus, Southampton SO17 1BJ, UK

1. **Continuity measurements:**

A new design was realized then another batch of samples was fabricated to characterize electrically the nanowires. Here, the nanowires do not have comb-like structure; instead each wire is accessible via two opposite electrodes to be able to measure the electrical continuity of the nanowires. The schematics, shown in Figure S1, present two electrical measurements experiments which were carried: first, the continuity of a single nanowire, where two probe-needles are connected to the same nanowire from both sides (shown in black). The linear current-voltage (IV) curve, for a DC voltage sweep from 0 V to 5 V then to -5 V and back to 0 V, demonstrates successful fabrication of continuous and conductive nanowires, confirming the results obtained by SEM observations. The second experiment (shown in blue) consists of checking the non-continuity (non-conductivity) of neighboring nanowires by connecting the two probe-needles to two adjacent nanowires then applying similar DC voltage sweeps. The blue IV curve shows extremely low current indicating that the adjacent nanowires are not connected (not touching), demonstrating successful fabrication of arrays of cross-points nanowires.

**
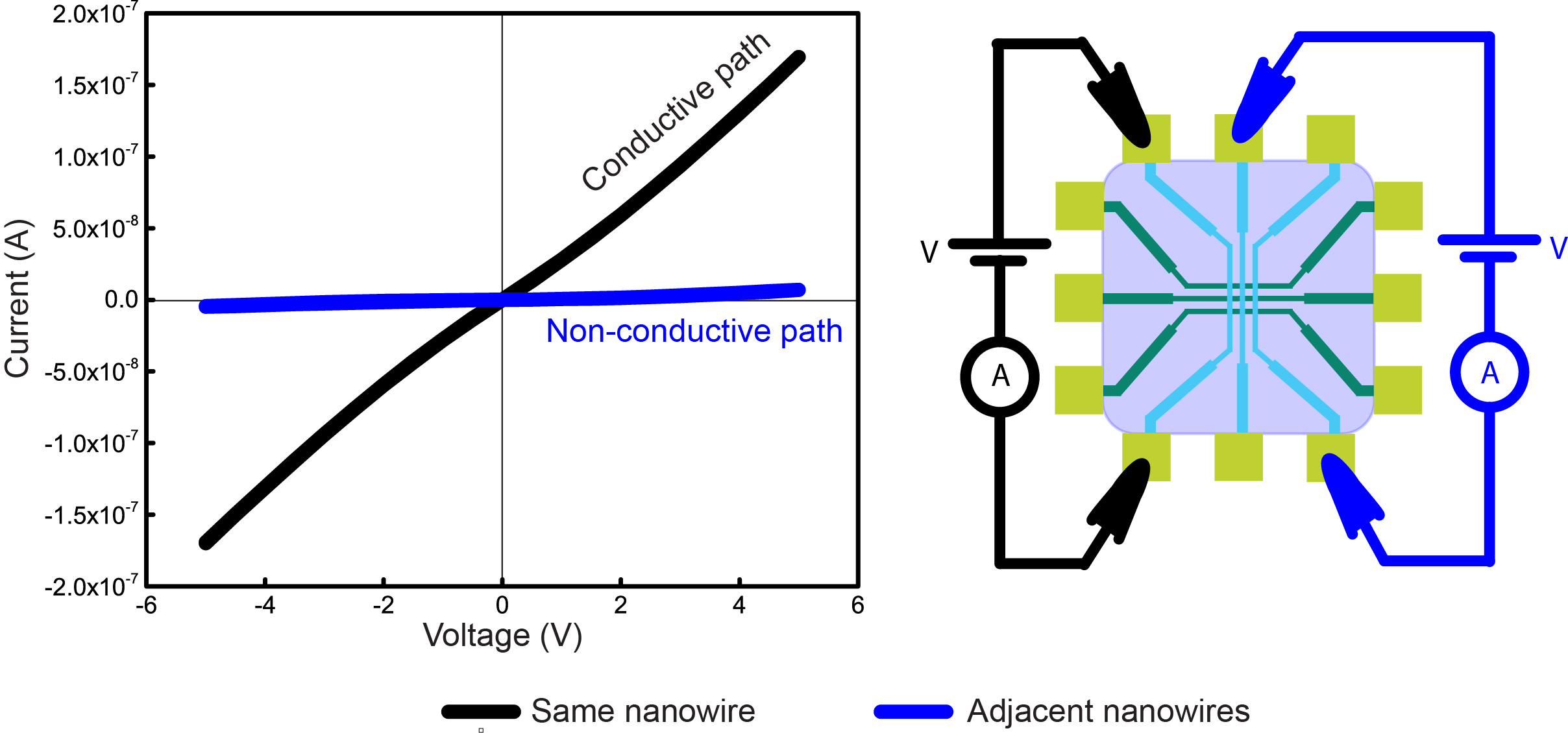
**

**Figure S1:** Continuity measurements of single nanowires. Connection schematics of continuous and adjacent nanowires, with their IV characteristics shown in black and blue colors, successively.

1. **Characterization of single memristor nano-devices:**

DC sweeping mode:

Figure S2 shows electrical characterizations of single memristive nano-devices. The results shown are obtained from 70×70 nm^2^ devices with the following stack TiN/TiO_2-x_/Pt/Ti (40nm/10nm/10nm/5nm). Top electrode (TiN) was connected the biasing voltage while the bottom electrode (Pt) was grounded. First, the device was electroformed with negative polarity, at -2 V, which brought the device from its high pristine resistive state to a much lower resistance. Then, four complete DC sweeping cycles [0, 5, -5, 0] V were realized that made the device toggle between low and high resistive states (LRS, HRS). Demonstrating promising and functional non-volatile nano-scale memristors.

**
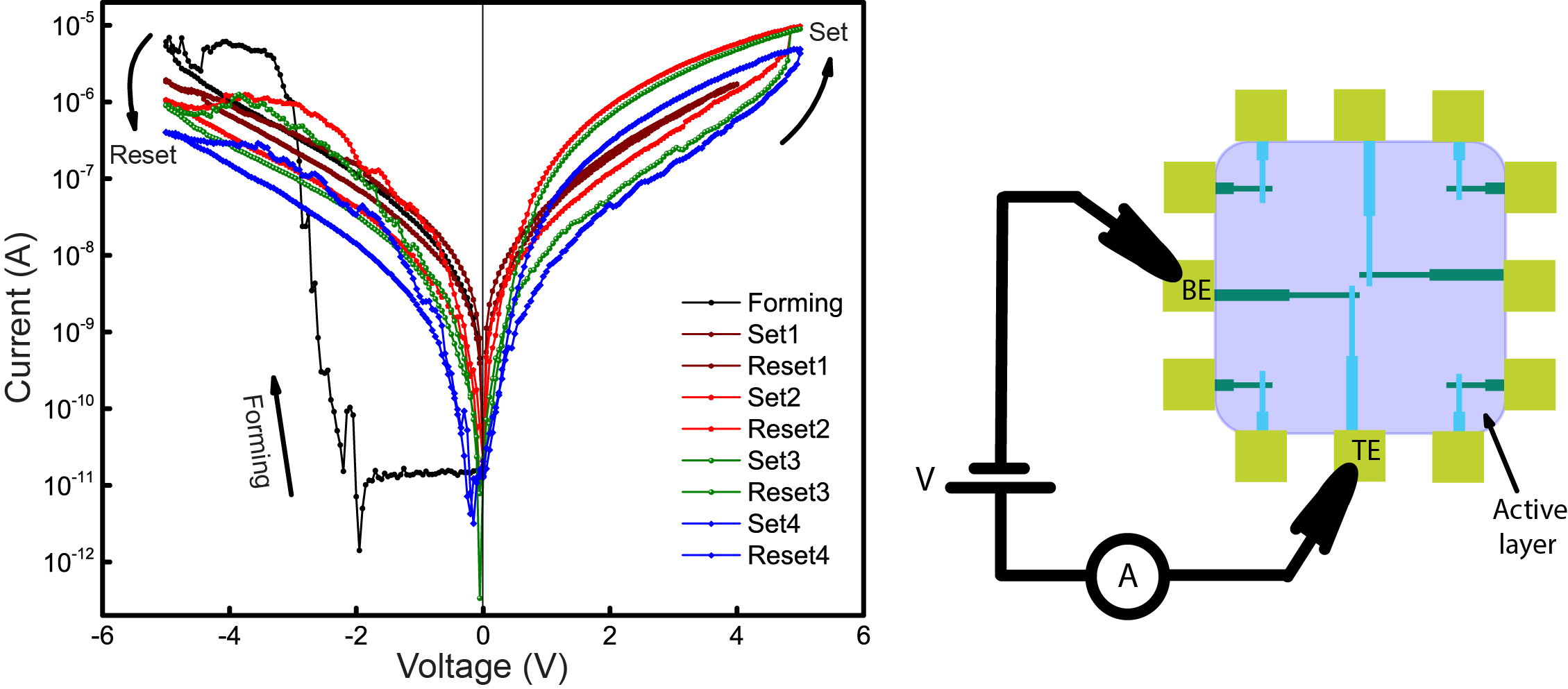
**

**Figure S2:** Electrical characterizations of single nano-device in DC sweeping mode. The current-voltage curves show electroforming step and four subsequent sweeping cycles. The schematic represents biasing method.

Pulsing mode:

Switching to slightly larger devices, 100×100 nm^2^, and biasing it in pulsing mode gives the results shown in Figure S3. In this experiment, the device was subject to trains of pulses with opposite polarities. In the first part, a train of pulses with gradual voltage increase (steps of ±0.1 V and pulse width of 5µs) of consecutive pulses with opposite polarities is applied. This step allows the device to switch to LRS or HRS (LRS in this case) in non-invasive fashion. The change in resistive state was fixed deliberately to 8%. Subsequently, trains of increasing pulses (0.1 V step) with one polarity at a time were applied. Reversing the pulsing polarity occurs when an 8% resistive state change has been achieved. Figure S3 shows 10 resistive state cycles between LRS and HRS achieved with an analogue behavior (gradual switching). All resistance measurements correspond to reading pulses taken at 0.2 V after each biasing pulse.

**
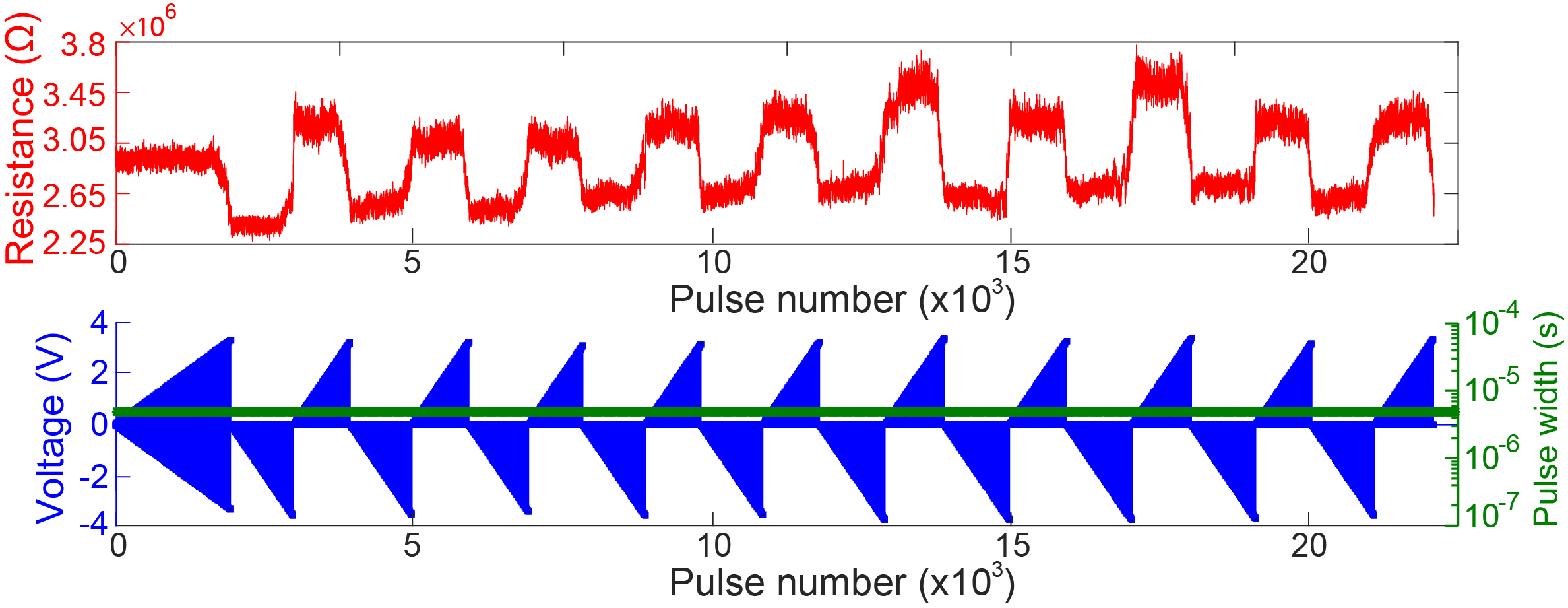
**

**Figure S3:** Electrical characterization of memristive nano-devices in pulsing mode. 10 consecutive gradual switching cycles are presented with 8% change in resistive state.

1. **Optimization of the device’s fabrication:**

Figure 4 was generated based on the results we have obtained from different configurations exposed in this paper. From SEM images we can see various results, such as the images shown in Figure S4 and S5:

a) SEM images of the 32 nanowires after liftoff of 15 nm wide nanowires with (a) 45 nm and (b) 30 nm gaps, using 40 nm thin diluted resist (Figure S4). Here, we distinguish well-developed features. These 2 images correspond to Figures 4.g and 4.h, respectively.

b) SEM images of the 32 nanowires after liftoff of 15 nm wide nanowires with 20 nm gaps, resulting into 4 possible results: (a) no nanowires, (b) well defined nanowires, (c) liftoff failure, and (d) merged features (Figure S5). These images correspond to Figure 4.i(1), Figure 4.i(2), Figure 4.i(3) and Figure 4.i(4), respectively.

From these observations we notice that successful resist development doesn’t mean successful liftoff (Figure S5.c).”

**
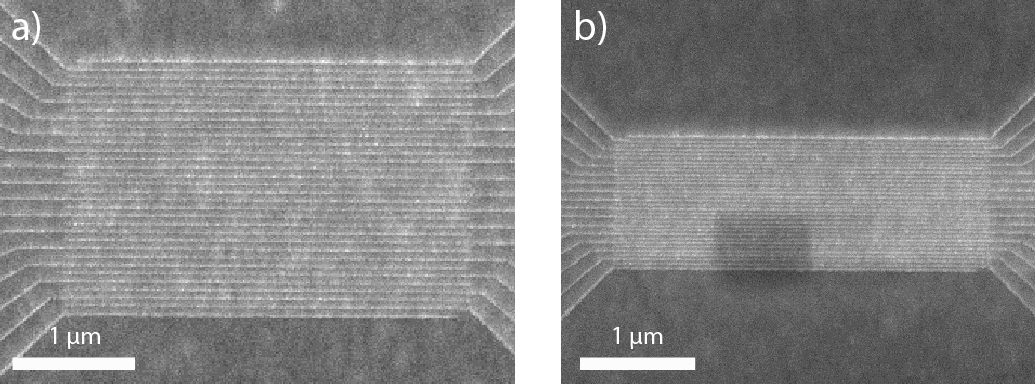
**

**Figure S4:** SEM images of Figure 4: (a) SEM image of the schematic shown in (a) Figure 4.g and (b) Figure 4.h, after liftoff process.

**
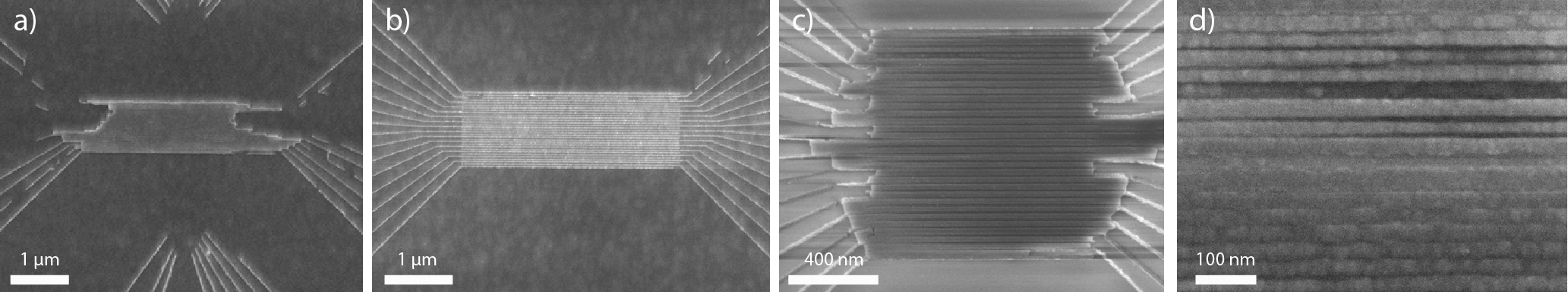
**

**Figure S5:** SEM images of the schematics shown in (a) Figure 4.i(1), (b) Figure 4.i(2), (c) Figure 4.i(3) and (d) Figure 4.i(4), after liftoff process.

**
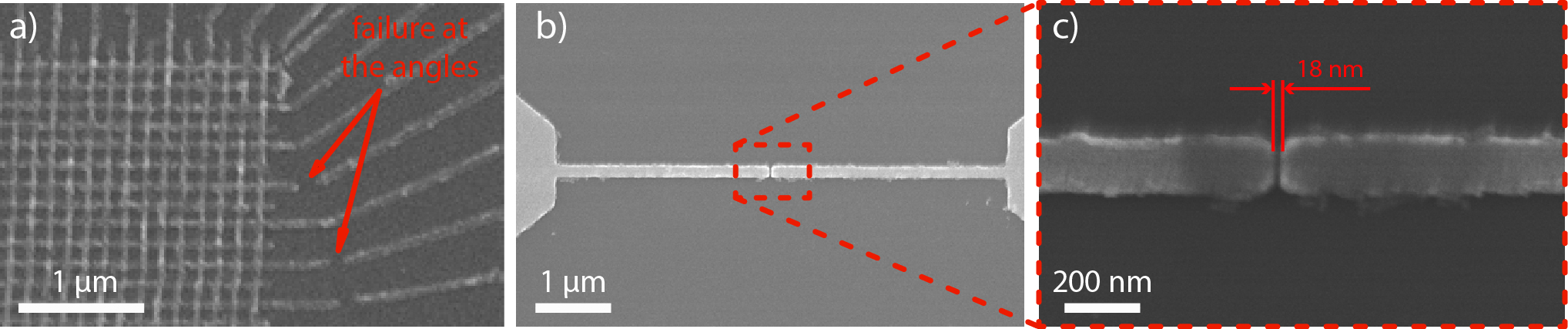
**

**Figure S6:** (a) Sub- 15 nm nanowires failure at the angles, resulting in low yield. (b) Planar devices, with 18 nm gap (c).

**
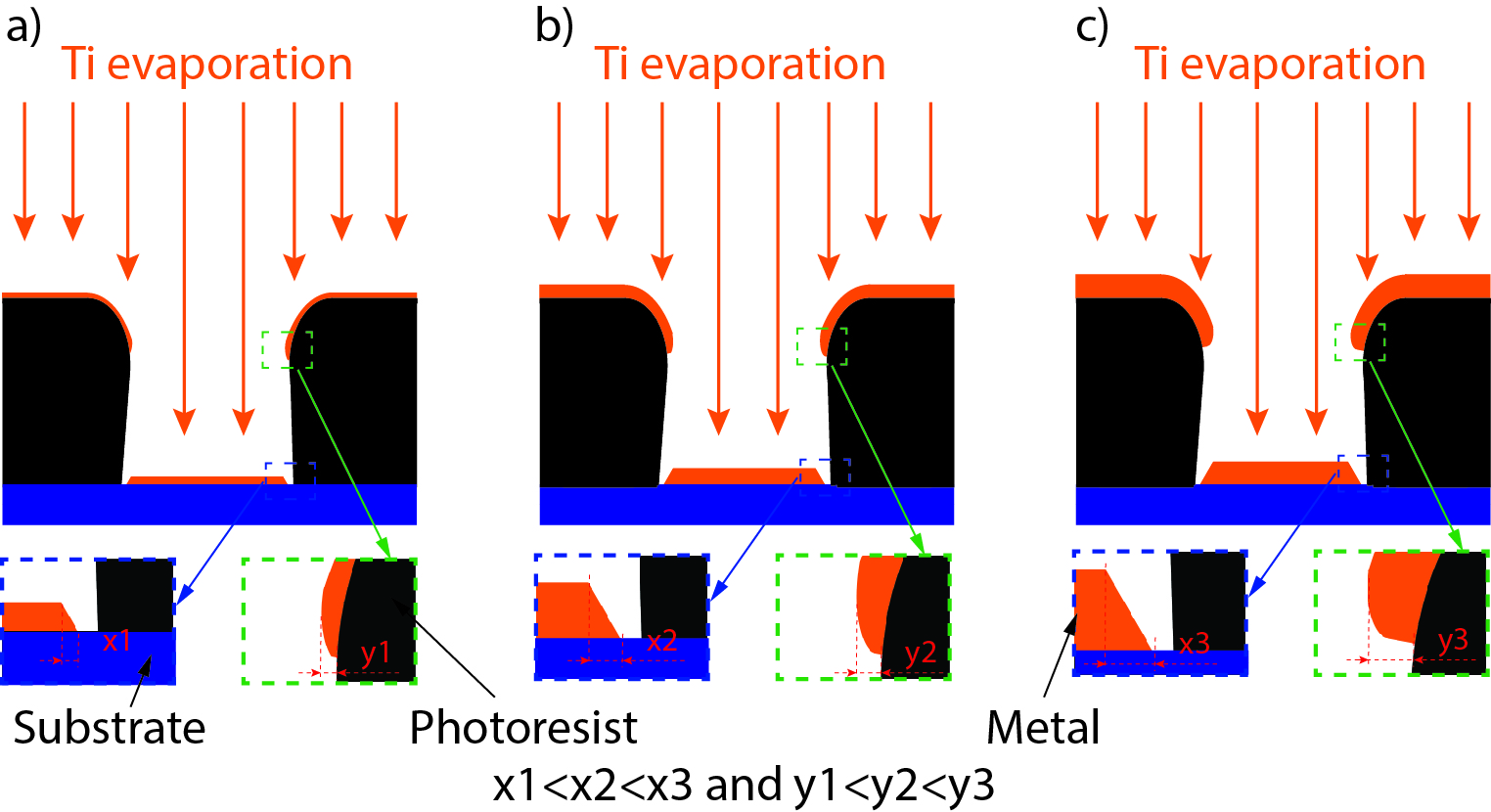
**

**Figure S7:** Nanowire’s profile during Ti deposition: (a) at the beginning (~3 nm) where metal, in orange, starts being deposited even on the resist edges (y1), which creates a slope in the wire edges deposited on the substrate, with a positive profile along x1, (b) at the mid-evaporation (7 nm) with y2>y1 which makes x2>x1, (c) at the end of evaporation (10 nm) where y3>y2 therefore x3 becomes larger (x3>x2).
